# Functional Improvement of Regulatory T Cells from Rheumatoid Arthritis Subjects Induced by Capsular Polysaccharide Glucuronoxylomannogalactan

**DOI:** 10.1371/journal.pone.0111163

**Published:** 2014-10-22

**Authors:** Eva Pericolini, Elena Gabrielli, Alessia Alunno, Elena Bartoloni Bocci, Stefano Perito, Siu-Kei Chow, Elio Cenci, Arturo Casadevall, Roberto Gerli, Anna Vecchiarelli

**Affiliations:** 1 Microbiology Section, Department of Experimental Medicine, University of Perugia, Perugia, Italy; 2 Rheumatology Unit, Department of Medicine, University of Perugia, Perugia, Italy; 3 Department of Microbiology and Immunology, Albert Einstein College of Medicine, New York, New York, United States of America; Stony Brook University, United States of America

## Abstract

**Objective:**

Regulatory T cells (Treg) play a critical role in the prevention of autoimmunity, and the suppressive activity of these cells is impaired in rheumatoid arthritis (RA). The aim of the present study was to investigate function and properties of Treg of RA patients in response to purified polysaccharide glucuronoxylomannogalactan (GXMGal).

**Methods:**

Flow cytometry and western blot analysis were used to investigate the frequency, function and properties of Treg cells.

**Results:**

GXMGal was able to: i) induce strong increase of FOXP3 on CD4^+^ T cells without affecting the number of CD4^+^CD25^+^FOXP3^+^ Treg cells with parallel increase in the percentage of non-conventional CD4^+^CD25^−^FOXP3^+^ Treg cells; ii) increase intracellular levels of TGF-β1 in CD4^+^CD25^−^FOXP3^+^ Treg cells and of IL-10 in both CD4^+^CD25^+^FOXP3^+^ and CD4^+^CD25^−^FOXP3^+^ Treg cells; iii) enhance the suppressive activity of CD4^+^CD25^+^FOXP3^+^ and CD4^+^CD25^−^FOXP3^+^ Treg cells in terms of inhibition of effector T cell activity and increased secretion of IL-10; iv) decrease Th1 response as demonstrated by inhibition of T-bet activation and down-regulation of IFN-γ and IL-12p70 production; v) decrease Th17 differentiation by down-regulating pSTAT3 activation and IL-17A, IL-23, IL-21, IL-22 and IL-6 production.

**Conclusion:**

These data show that GXMGal improves Treg functions and increases the number and function of CD4^+^CD25^−^FOXP3^+^ Treg cells of RA patients. It is suggested that GXMGal may be potentially useful for restoring impaired Treg functions in autoimmune disorders and for developing Treg cell-based strategies for the treatment of these diseases.

## Introduction

Rheumatoid arthritis (RA) is an autoimmune disease characterized by destructive joint inflammation. The synovial inflammation is characterized by non-specific infiltration of both lymphocytes and innate immune cells, such as synoviocytes, macrophages and neutrophils [1]. RA is generally considered to be an autoimmune disease in which pathogenic T cells such as T helper (Th)1 and Th17 cells play an important role [2]. An exciting aspect of Th17 cell homeostasis is the reciprocal relationship with regulatory T cells (Treg), whose imbalance is believed to play a major role in the development of autoimmune disease [3]–[6]. Treg were originally identified by high surface expression of CD25 [7], [8] and subsequently by the forkhead box protein P3 (FOXP3) transcriptional factor, which controls their development and suppressive function [9], [10]. Interestingly, it was suggested that activated FOXP3^+^ non-Treg cells may be a reservoir of silent Treg that regain their function following activation [11]. The data on the effective number of Treg cells in RA patients are inconsistent. Some studies report a decreased number of Treg cells in the blood of RA [12], while others report increased numbers of Treg cells [13], however it is clear that Treg cells appear unable to suppress inflammation in the rheumatoid joints [14]. Thus, paradoxically, CD4^+^CD25^+^ T cells, including CD4^+^CD25^+^FOXP3^+^ Treg cells, circulate in RA patients despite the still ongoing inflammation [13], [14]. The capacity of Treg cells to suppress several arthritic responses both in humans and animal models makes them potential therapeutic targets in arthritic conditions such as RA [15]. A prior study suggested that the therapeutic efficacy of methotrexate (MTX), the current “gold standard” treatment in experimental RA, was partly attributed to the increased development of CD4^+^CD25^+^ Treg cells [16]. Furthermore, anti-rheumatic biotechnological therapies may offer a means of restoring the Th17/Treg cell balance in favor of Treg, thereby re-establishing immune tolerance [6]. It has been demonstrated that some fungal products, such as those from *Cryptococcus neoformans* (*C. neoformans*), directly induce lymphocytes apoptosis [17]. Accordingly, we demonstrated that glucuronoxylomannogalactan (GXMGal), a purified capsular polysaccharide from the opportunistic fungus *C. neoformans* [18], directly induce apoptosis of activated lymphocytes [19]. This was recently evidenced also using activated RA T cells producing IL-17A suggesting the potential role of GXMGal in fighting deleterious Th17 cells [20]. In light of this finding, the aim of this study was to understand whether GXMGal could restore the Th17/Treg balance, both by dampening Th17 response [20] and modulating Treg cell suppressive capacity.

## Methods

### Cryptococcal polysaccharide and other reagents

RPMI-1640 with L-glutamine, FCS and penicillin streptomycin solution were obtained from EuroClone S.p.A. (Milan, Italy). Brefeldin A, PHA, dexametasone (DEX), MTX, the inhibitor of pSTAT3 (FLLL31) and hypotonic propidium iodide solution (PI) were obtained from Sigma-Aldrich (St. Louis, MO, USA). Monoclonal antibodies (mAbs) to human CD3 and CD28 were obtained from ImmunoTools GmbH (Friesoythe, Germany).

GXMGal [21], known until recently as galactoxylomannan, was purified as described elsewhere [22], [23]. The dose of GXMGal was chosen on the basis of our previous published data [24]. All reagent and media were negative for endotoxin, as assessed by *Limulus* amebocyte lysate assay (QCL-1000, BioWhittaker, VWR International p.b.i., Milan, Italy).

### Subjects

Thirty patients with established RA according to the American College of Rheumatology classification criteria [25] and followed up at the Rheumatology Unit of the Perugia University, were included in the study. This cohort included 20 women and 10 men with a mean age of 54±8 years (mean ± SD) and disease duration of 12±6 years. The mean Disease Activity Score, as evaluated including 28 swollen and tender joint count (DAS28), was 3±1.2 at the time of sample collection. All patients were MTX naïve and were receiving other synthetic or biologic disease-modifying anti-rheumatic drugs in monotherapy (leflunomide: n° 11, hydroxychloroquine: n° 16, tocilizumab: n° 3). Fourteen patients were also receiving low doses of corticosteroids (5 mg/day of prednisone or equivalent). Fifteen healthy subjects matched for age and sex acted as normal control (Control). Written informed consent was obtained from all subjects prior to sample collection in accordance with the Declaration of Helsinki. Local Ethical Committee CEAS (Comitato Etico delle Aziende Sanitarie, Umbria, Italy) approval was received for the whole study (AR-2 n. 1874/12).

### Isolation of cells and culture condition

Heparinized venous blood was obtained from Control and RA patients. Peripheral blood mononuclear cells (PBMC) were separated by density gradient centrifugation on Ficoll-Hypaque (EuroClone). PBMC were incubated with mAb-conjugated MicroBeads to human CD4 (Miltenyi Biotec) and CD4^+^ T lymphocytes were purified by magnetic separation. Purity of separated CD4^+^ T cells was >90% as detected by cytofluorimetric analysis. CD4^+^CD25^+^ and CD4^+^CD25^−^ cells were purified from CD4^+^ T lymphocytes using the CD4^+^CD25^+^ Treg cell isolation kit (Miltenyi Biotec) according to manufacturer's instructions. Unless otherwise specified, cells were seeded in 48 or 96-well culture plates pre-coated with mAbs to CD3 plus CD28 (both 2 µg/ml) and activated for 30 min with soluble mAbs to CD3 plus CD28 (both 3 µg/ml) (ImmunoTools) in RPMI-1640+10% FCS (complete medium) at 37°C plus 5% CO_2_. After activation cells were washed and stimulated as described below.

### Cytokine Production

Activated PBMC (5×10^6^/ml) were incubated for 2, 18 and 72 h in the presence or absence of GXMGal (10 µg/ml), MTX (10 ng/ml) [26], DEX (10 nM) [27] or FLLL31 (5 µM) [20]. The supernatants were collected and cytokines were tested by specific ELISA assays (all from eBioscience, San Diego, CA). Cytokine titers were calculated relative to standard curves.

### Western Blot Analysis

Activated PBMC or CD4^+^ T cells (both 5×10^6^/ml) were incubated for different times in the presence or absence of GXMGal (10 µg/ml), MTX (10 ng/ml), DEX (10 nM) or FLLL31 (5 µM). After incubation, proteins were extracted and subjected to western blotting as previously described [20]. The membranes were incubated overnight with polyclonal Abs to pSTAT3 (goat; Tyr 705), STAT3 (rabbit), FOXP3 (rabbit), T-bet (rabbit) (all dilution 1/200; all Santa Cruz Biotechnology, Delaware Avenue, Santa Cruz, CA) in blocking buffer. Immunoreactive bands were visualized and quantified by Chemidoc Instrument (Bio-Rad Laboratories, Hercules, CA) [28].

### Flow Cytometry Analysis

Activated PBMC (1×10^6^/ml) were incubated for 2 or 18 h in the presence or absence of GXMGal (10 µg/ml) or MTX (10 ng/ml). After incubation, cells were fixed with 1.5% formalin for 10 min at room temperature (RT), washed and incubated with FITC-labelled mAb to human CD4, APC-labelled mAb to human CD25 (both mouse IgG1 isotype; 2 µl/tube; ImmunoTools) or PerCP-Cy5.5 labelled mAb to human CD127 (mouse IgG1, 5 µl/tube; eBioscience) for 20 min at RT. After incubation, cells were treated as previously described [29] and incubated with PE-labelled mAb to human FOXP3 (mouse IgG1 isotype; 20 µl/tube) (BD Biosciences, San Jose, CA). In selected experiments, activated purified Treg (1×10^6^/ml) from RA were treated or not with GXMGal (10 µg/ml) for 18 h and then incubated with PE-labelled mAb to human FOXP3 (mouse IgG1 isotype; 20 µl/tube) (BD Biosciences) for 20 min on ice. After incubation, cells were analyzed by flow cytometry using FACSCalibur (BD Biosciences). Data are expressed as percentage of positive cells or as mean of fluorescence intensity (MFI). Autofluorescence and control staining were evaluated as previously described [29].

### Intracellular staining

Activated purified CD4^+^ T cells (1×10^6^/ml) were incubated for 18 or 96 h in the presence or absence of GXMGal (10 µg/ml) or MTX (10 ng/ml). Brefeldin A (10 µg/ml) was added 2 h after GXMGal or MTX stimulation [30]. After incubation, cells were fixed with 1.5% formalin for 10 min at RT, washed and incubated with APC-labelled mAb to human CD25 (mouse IgG1 isotype; 2 µl/tube; ImmunoTools) for 20 min at RT. After incubation, cells were treated as previously described [29] and incubated with PE-labelled mAb to human FOXP3 (mouse IgG1 isotype; 20 µl/tube) (BD Biosciences), with purified mAbs to human IL-10 (mouse IgG2b isotype, 2 µg/ml) or TGF-β1 (mouse IgG1 isotype, 25 µg/ml) (both Sigma-Aldrich) [29] or in selected experiments with FITC-labelled mAb to human IFN-γ (mouse IgG_2b_ isotype; 10 µl/tube) (Sigma-Aldrich) and with Ab to T-bet (rabbit) (1/50; Santa Cruz) for 20 min on ice followed by Cy3 labelled conjugated affinity purified secondary antibody (dil. 1/250; Chemicon Int.). Autofluorescence and control staining were evaluated as previously described [29].

### Apoptosis

Naïve or activated CD4^+^CD25^+^ Treg cells were stimulated in the presence or absence of GXMGal (10 µg/ml) or MTX (10 ng/ml). The percentage of cells undergoing apoptosis was evaluated by flow cytometry analysis as previously described [29].

### CFSE-based suppression assays

CD4^+^ responder T cells (Tresp) (1×10^6^/ml) were stained with CFSE (1 µM) (Sigma-Aldrich) according to the manufacturer's instructions [31] and then seeded in 96-well culture plates pre-coated with mAbs to CD3 plus CD28 (both 2 µg/ml) and activated for 30 min with PHA (10 µg/ml) (Sigma-Aldrich) at 37°C plus 5% CO_2_ in complete medium. Cells were then washed and co-cultured for 96 h in the presence or absence of autologous CD4^+^CD25^+^ Treg cells (Tresp/Treg: 16/1) that have been pre-treated for 18 h in the presence or absence of GXMGal (10 µg/ml) or MTX (10 ng/ml). After 96 h the suppressive activity of CD4^+^CD25^+^ Treg cells was evaluated by measuring the percentage of inhibition of proliferation of CD4^+^ Tresp cells by flow cytometry analysis [32]. After 96 h the percentage of CFSE-stained CD4^+^CD25^+^ Treg cells proliferation was determined as above described.

### Statistical analysis

The results reported in the bar graphs are the mean ± SEM from triplicate samples of 5–10 different RA patients or Controls. Mann-Whitney U test was used for statistical analysis, except for the determination of CD25^+^FOXP3^+^ and CD25^−^FOXP3^+^ cells in which the statistic was calculated according to Student's t test.

A value of *p*<0.05 was considered significant.

## Results

**Figure 1:**
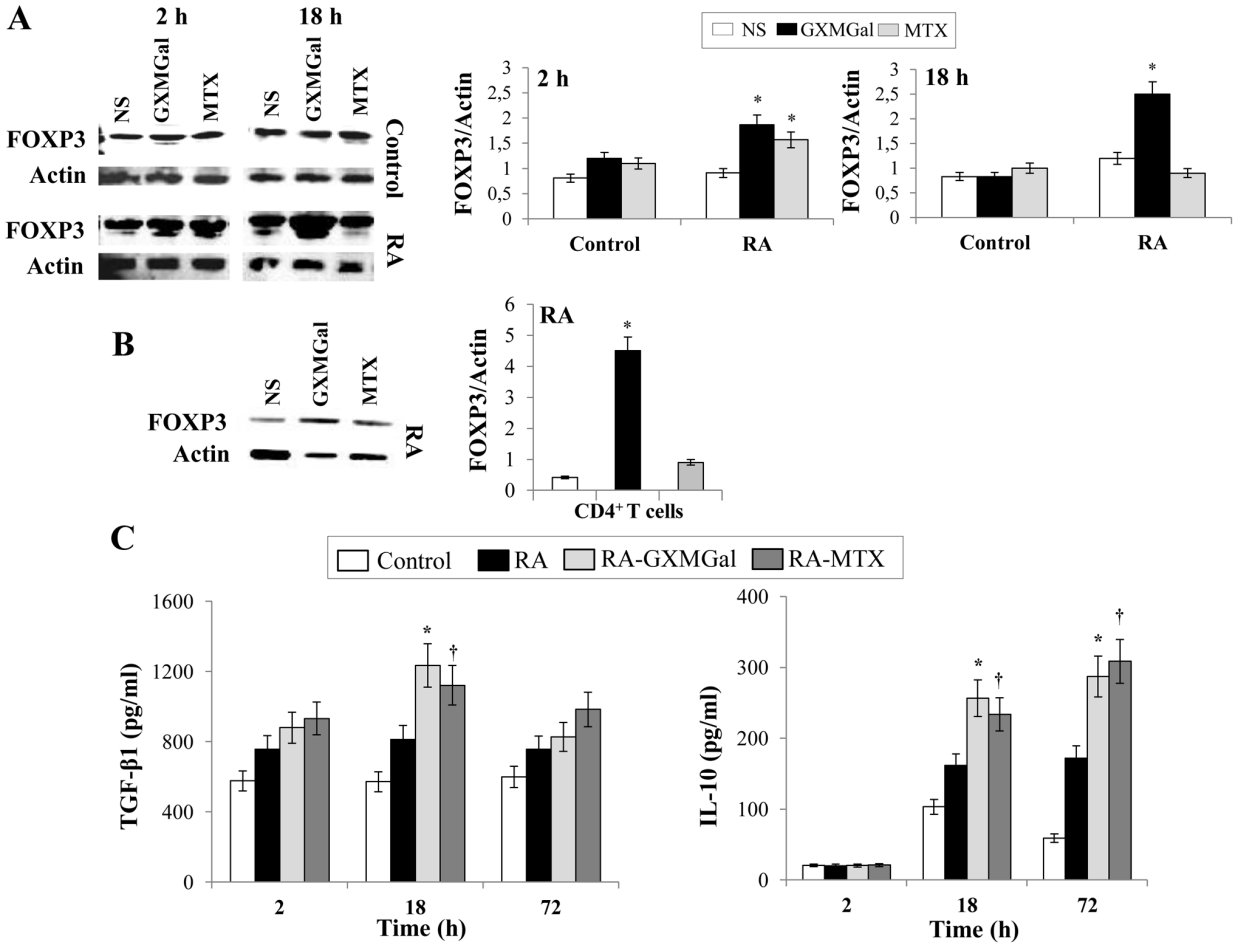
GXMGal effect on Treg cell response. Activated PBMC (**A** and **C**) or purified CD4^+^ T cells (**B**) (both 5×10^6^/ml) from Control and RA were incubated for 2, 18 and 72 h in the presence or absence (NS) of GXMGal (10 µg/ml) or MTX (10 ng/ml). After 2 and 18 h (**A**) or 18 h (**B**) of incubation, cell lysates were analyzed by western blotting. Membranes were incubated with Ab to FOXP3. Actin was used as an internal loading control. Normalization was shown as mean ± SEM of five independent experiments (**A** and **B**). *, *p*<0.05 (triplicate samples of 5 different Control and RA; RA treated *vs* untreated cells). Culture supernatants were collected after 2, 18 and 72 h to test TGF-β1 and IL-10 levels by specific ELISA assays. *, *p*<0.05 (triplicate samples of 7 different Control and RA; RA GXMGal-treated *vs* untreated cells); ^†^, *p*<0.05 (triplicate samples of 7 different Control and RA; RA MTX-treated *vs* untreated cells) (**C**).

Firstly, we analyzed the effect of GXMGal on FOXP3 expression in PBMC and CD4^+^ T cells from RA patients. PBMC from RA patients and from healthy donors (Control) were treated with GXMGal or MTX for 2 and 18 h and the expression of FOXP3 was evaluated. The results showed that GXMGal markedly induced FOXP3 activation after 2 and 18 h of incubation, while MTX only after 2 h (Fig. 1A). GXMGal and MTX treatment did not produce any modulation on PBMC from Control. GXMGal-induced FOXP3 activation at 18 h was also confirmed by using purified CD4^+^ T cells from RA (Fig. 1B). Furthermore, we analyzed key cytokines such as TGF-β1 and IL-10 involved in Treg activation [33]. To this purpose PBMC were treated with GXMGal for 2, 18 and 72 h and the possible modulation of TGF-β1 and IL-10 production was tested. Both cytokines were produced at higher levels in culture supernatants of PBMC from RA patients with respect to Control. The production of TGF-β1 and IL-10 was further enhanced by GXMGal treatment after 18 h or 18 and 72 h of incubation respectively (Fig. 1C). The lack of the increased production of TGF-β1 after 72 h of incubation could be due to the reutilization of this cytokine by the cells during the incubation period. MTX produced similar effects.

**Figure 2:**
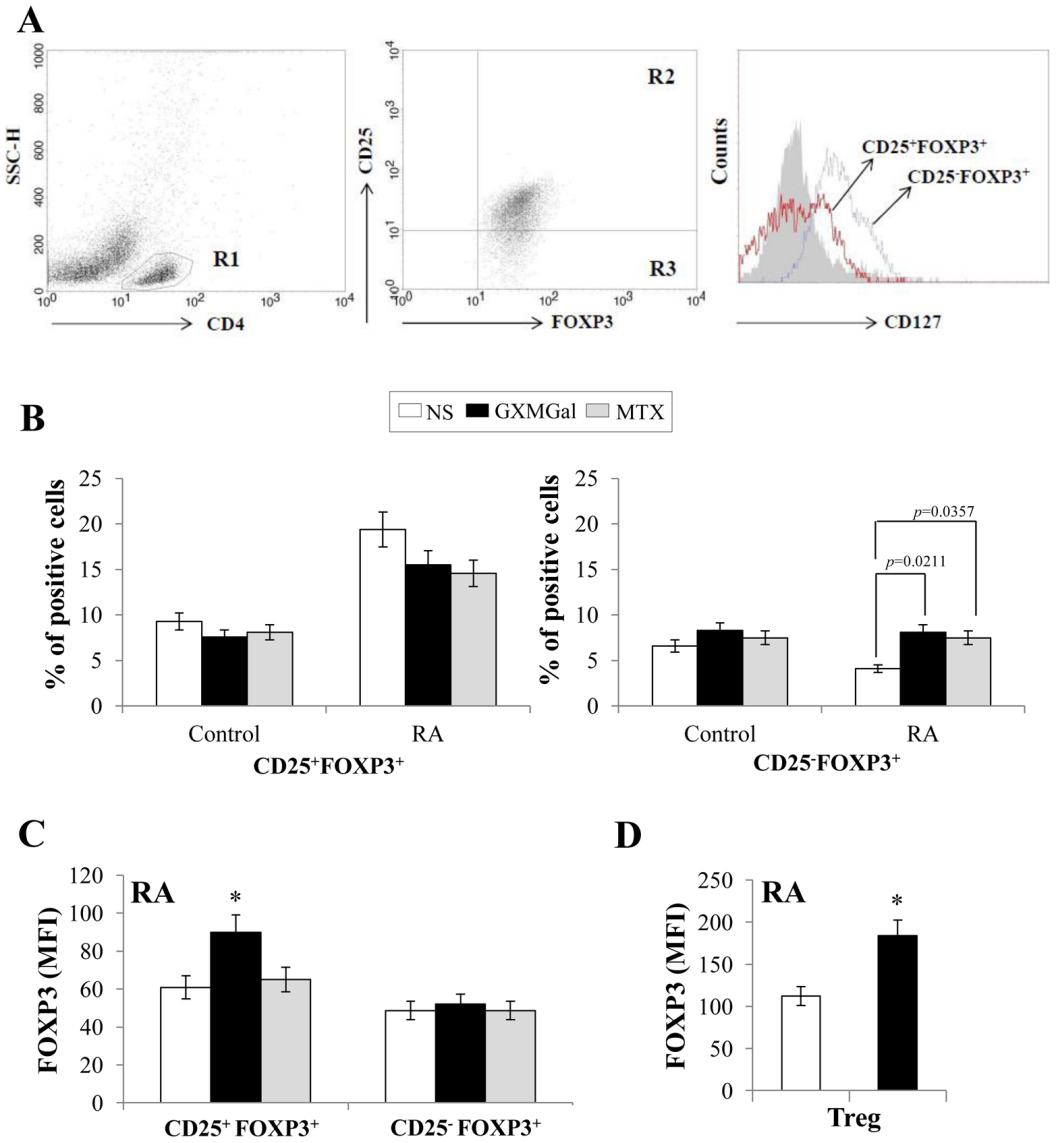
GXMGal effect on different subsets of Treg cells. Activated PBMC (1×10^6^/ml) from Control and RA were incubated for 2 h in the presence or absence (NS) of GXMGal (10 µg/ml) or MTX (10 ng/ml). After incubation, cells were stained for cell surface expression of CD4, CD25 and CD127 and then intracellular stained for FOXP3. During the acquisition step a population of PBMC enriched of CD4^+^ T cells was obtained. For analysis, the CD4^+^ lymphocytes were gated on PBMC (based on side light scatter and CD4 staining: R1) and analyzed for CD25 and FOXP3 expression (CD25^+^FOXP3^+^: R2 and CD25^−^FOXP3^+^: R3). The expression of CD127 was shown as FACS histograms in R2 and R3 cells. The gating strategy was shown (**A**). The percentage of CD25^+^FOXP3^+^ and CD25^−^FOXP3^+^ cells are shown as mean ± SEM of ten independent experiments. *p* = 0.0211 (triplicate samples of 10 different Control and RA; RA GXMGal-treated *vs* untreated cells); *p* = 0.0357 (triplicate samples of 10 different Control and RA; RA MTX-treated *vs* untreated cells (**B**). The mean of fluorescence intensity (MFI) of FOXP3 in CD25^+^FOXP3^+^ and CD25^−^FOXP3^+^ cells (**C**) or magnetically purified Treg (**D**) from RA after 18 h of GXMGal or MTX treatment was shown as mean ± SEM of five independent experiments. *, *p*<0.05 (triplicate samples of 5 different RA; RA GXMGal-treated *vs* untreated cells).

The proportion of conventional Treg, identified by the expression of CD25 molecule and FOXP3 (CD25^+^FOXP3^+^), and non-conventional Treg CD25^−^FOXP3^+^ was tested after GXMGal treatment in PBMC from RA patients and Control. CD4^+^ T cells were gated on PBMC and analyzed for CD25, FOXP3 and CD127 [33] expression (Fig. 2A). It is known that Treg CD25^+^FOXP3^+^ are CD127^low^ [34]. As shown in Figure 2B the treatment with GXMGal for 2 h did not modulate the percentage of CD25^+^FOXP3^+^ Treg; however, GXMGal increased the percentage of non-conventional CD25^−^FOXP3^+^ Treg. The prolonged treatment with GXMGal (18 h) did not modulate the percentage of CD25^+^FOXP3^+^ or CD25^−^FOXP3^+^ Treg (not shown). Similar effect was observed by using MTX (Fig. 2B). GXMGal-induced FOXP3 expression at 18 h was evidenced in CD25^+^FOXP3^+^ but not in CD25^−^FOXP3^+^ Treg from RA (Fig. 2C). This was confirmed by using magnetically purified Treg from RA (Fig. 2D). Given that we previously demonstrated that GXMGal is able to induce apoptosis of activated T cells [19], [29], we evaluated whether GXMGal affects apoptosis of Treg. Our results showed that treatment with GXMGal or MTX did not modulate neither apoptosis of naïve and activated RA Treg nor their proliferation (data not shown).

**Figure 3:**
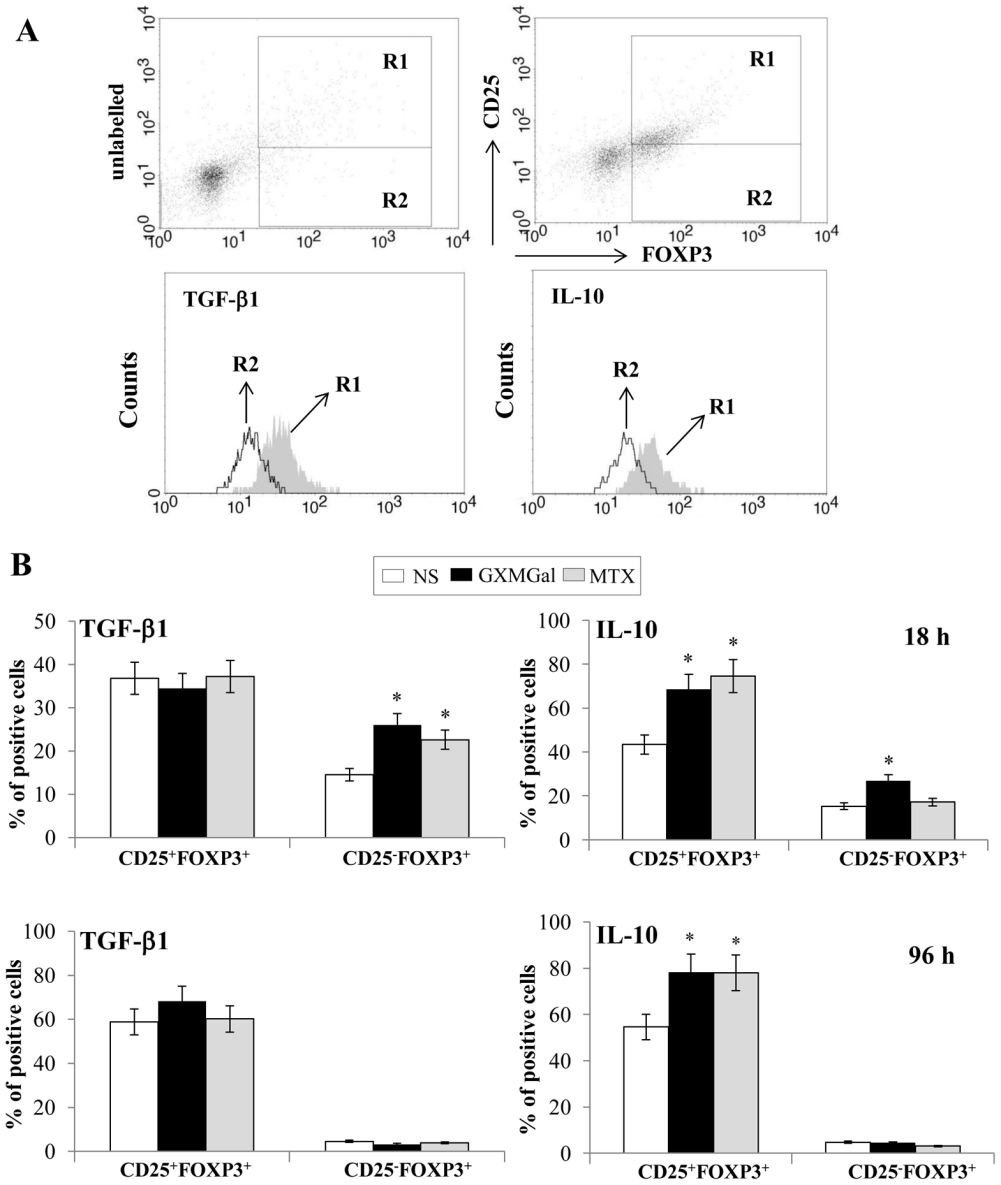
GXMGal effect on Treg cell intracellular cytokines production. Activated purified CD4^+^ T cells (1×10^6^/ml) from RA were incubated for 18 or 96 h in the presence or absence (NS) of GXMGal (10 µg/ml) or MTX (10 ng/ml). After incubation, cells were stained for cell surface expression of CD25 then intracellular stained for FOXP3 and TGF-β1 or IL-10. For the analysis of intracellular cytokines, CD25^+^FOXP3^+^ (R1) and CD25^−^FOXP3^+^ (R2) cells were gated on purified CD4^+^ T cells. The gating strategy was shown (**A**). The percentage of intracellular TGF-β1 and IL-10 on CD25^+^FOXP3^+^ and CD25^−^FOXP3 positive cells were shown after 18 h or 96 h (**B**) as mean ± SEM of ten independent experiments. *, *p*<0.05 (triplicate samples of 10 different RA; treated *vs* untreated cells).

**Figure 4:**
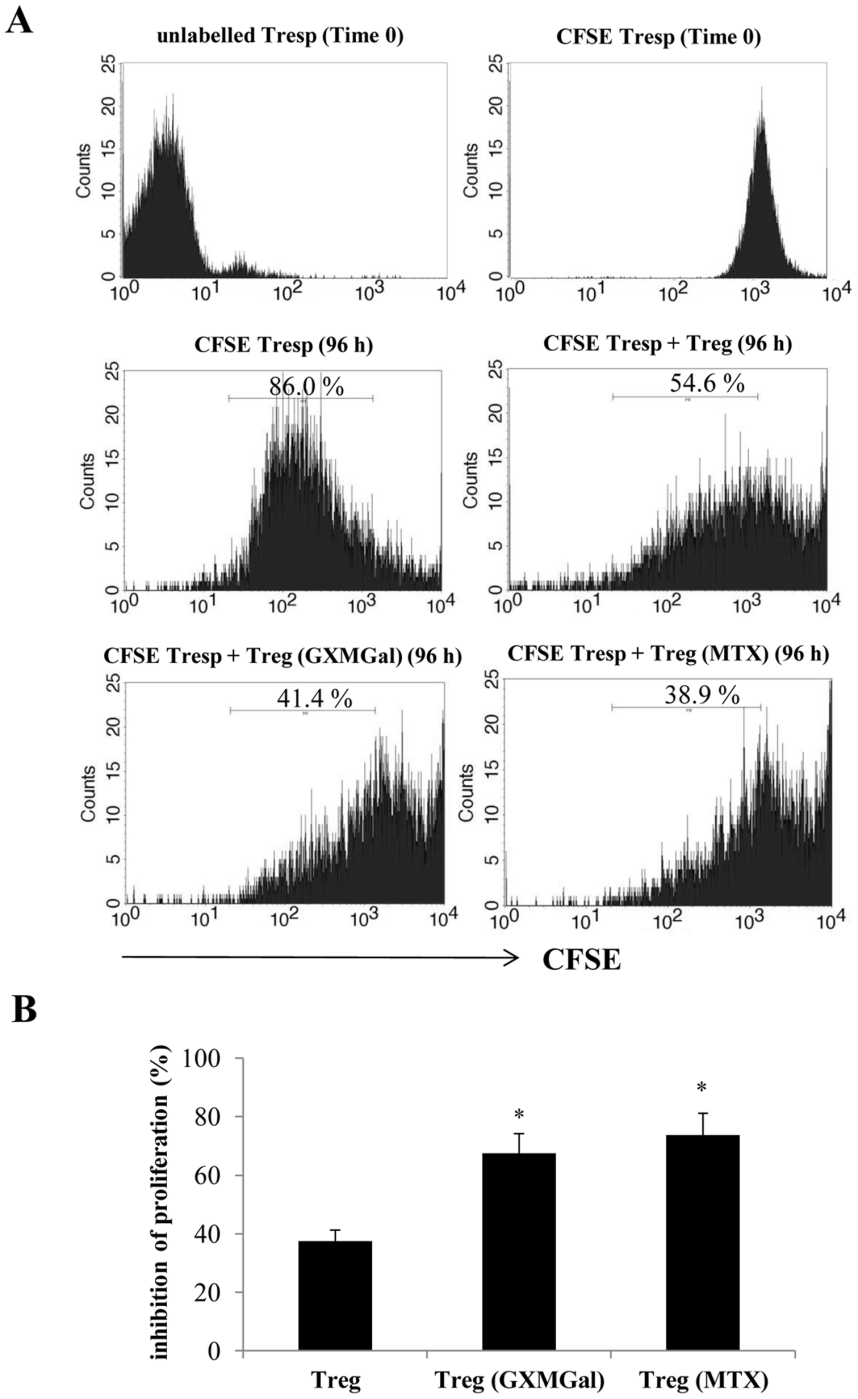
GXMGal effect on Treg cell suppressive activity. Activated RA purified CD4^+^ T cells (1×10^6^/ml) were stained with CFSE (1 µM) and then co-cultured for 96 h in the presence or absence of magnetically purified autologous RA Treg cells (Tresp/Treg: 16/1) that have been pre-treated for 18 h in the presence or absence of GXMGal (10 µg/ml) or MTX (10 ng/ml). After 96 h the suppressive activity of Treg cells was evaluated by measuring the percentage of inhibition of proliferation of CD4^+^ responder T cells (Tresp). Representative CFSE histograms show the distribution of proliferating CFSE-labelled Tresp according to the intensity of the CFSE label from the start of the experiment (Time 0) until 96 h. Given that the initial cell labelling is fairly homogeneous, each CFSE peak represents a cohort of cells that proceed synchronously through the division rounds. The areas within each histograms delimitated by the marker represent the percentage of divided CFSE-labeled cells (**A**). Mean ± SEM of percentage of proliferation inhibition is shown as bar graph (**B**). *, *p*<0.05 (triplicate samples of 10 different RA; treated *vs* untreated cells).

**Figure 5:**
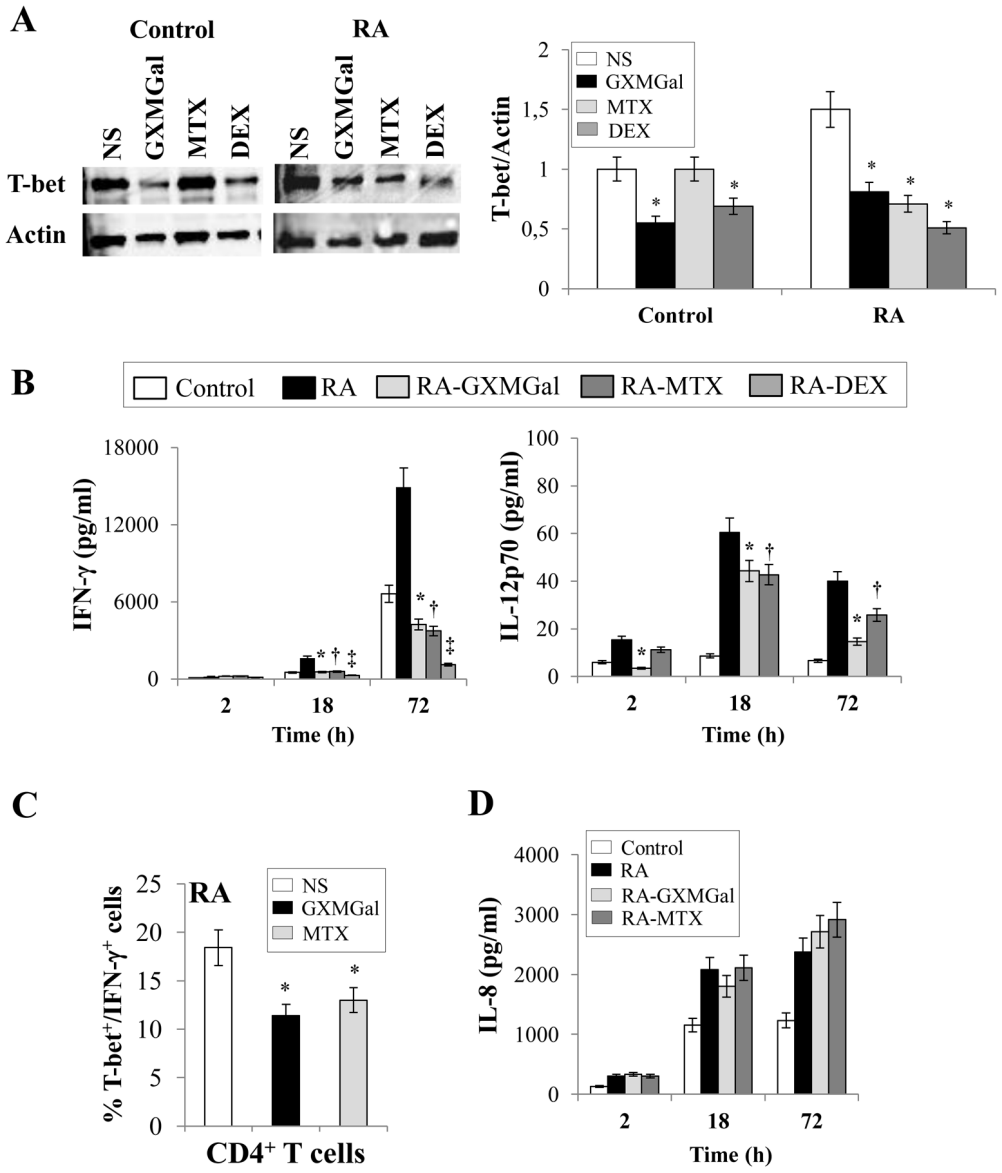
GXMGal effect on Th1 response. Activated PBMC (**A**, **B** and **D**) (5×10^6^/ml) from Control and RA were incubated for 20 min, 2, 18 and 72 h in the presence or absence (NS) of GXMGal (10 µg/ml), MTX (10 ng/ml) or DEX (10 nM). After 20 min of incubation, cell lysates were analyzed by western blotting. Membranes were incubated with Ab to T-bet. Actin was used as an internal loading control. Normalization was shown as mean ± SEM of five independent experiments. *, *p*<0.05 (triplicate samples of 5 different Control and RA; treated *vs* untreated cells) (**A**). Culture supernatants were collected after 2, 18 and 72 h to test IFN-γ, IL-12p70 (**B**) and IL-8 (**D**) levels by specific ELISA assays. *, *p*<0.05 (triplicate samples of 7 different Control and RA; RA GXMGal-treated *vs* untreated cells); ^†^, *p*<0.05 (triplicate samples of 7 different Control and RA; RA MTX-treated *vs* untreated cells); ‡, *p*<0.05 (triplicate samples of 7 different Control and RA; RA DEX-treated *vs* untreated cells). Activated purified CD4^+^ T cells (1×10^6^/ml) (**C**) from RA were stimulated as above described and intracellular stained for T-bet and IFN-γ. The percentage of T-bet^+^/IFN-γ^+^ CD4^+^ T cells from RA after 18 h of GXMGal or MTX treatment was shown as mean ± SEM of five independent experiments. *, *p*<0.05 (triplicate samples of five different RA; treated *vs* untreated cells).

Given that TGF-β1 and IL-10 production was determined in PBMC supernatants (Fig. 1C), we analyzed the level of these cytokines in both CD25^+^FOXP3^+^ and CD25^−^FOXP3^+^ cells. For the analysis of intracellular cytokines, CD25^+^FOXP3^+^ (R1) and CD25^−^FOXP3^+^ (R2) were gated on magnetically purified CD4^+^ T cells (Fig. 3A). After 18 h of incubation, both GXMGal and MTX induced a significant increase of intracellular TGF-β1 production in purified CD25^−^FOXP3^+^ cells, but they did not modulate this cytokine in purified CD25^+^FOXP3^+^ cells (Fig 3B). Moreover, intracellular IL-10 was significantly increased by GXMGal stimulation in both CD25^+^FOXP3^+^ and CD25^−^FOXP3^+^ cells. MTX induced a significant increase of intracellular IL-10 only in CD25^+^FOXP3^+^ cells (Fig. 3B). Moreover, when the treatment with GXMGal or MTX was prolonged till 96 h, a persistence of intracellular increase of IL-10 was detected only in CD25^+^FOXP3^+^ cells. At this time, no modulation of TGF-β1 was observed in both CD25^+^FOXP3^+^ and CD25^−^FOXP3^+^ cells (Fig. 3B). Moreover, we verified the *in vitro* functional activity of Treg towards autologous responder T cells (Tresp). RA activated CFSE-labelled Tresp were cultured for 96 h in the presence or absence of autologous Treg pre-treated for 18 h with or without GXMGal or MTX. Our data demonstrated that proliferation of RA Tresp cells was suppressed by Treg and this effect was significantly potentiated when Treg cells were pre-treated with GXMGal or MTX (Fig. 4A and B). Since it is recognized that Th1 cells play an important pro-inflammatory role in the pathogenesis of RA, the effect of GXMGal on Th1 differentiation was also evaluated. PBMC from RA patients and Control were treated with GXMGal, MTX and DEX for 20 min. It is well established that DEX can inhibit Th1 activation [27] and therefore DEX was used as inhibitor of T-box-containing protein expressed in T cells (T-bet) activation, exclusively expressed in Th1 cells. The results showed that GXMGal treatment was able to significantly suppress T-bet activation in PBMC from RA and Control. A similar effect was observed with DEX, whereas MTX produced inhibitory effects only in PBMC from RA patients (Fig. 5A). To further investigate the role of GXMGal in Th1 response, we analyzed the production of IFN-γ and IL-12p70, that were produced at higher levels in PBMC culture supernatants from RA compared to Control, after 18 and 72 h or 2, 18 and 72 h, respectively (Fig. 5B). A significant inhibition of IFN-γ production was observed after treatment with GXMGal or MTX after 18 and 72 h of incubation. As expected, DEX significantly reduced IFN-γ production (Fig. 5B). IL-12p70 was significantly inhibited by GXMGal at all times tested and by MTX after 18 and 72 h of incubation (Fig. 5B). GXMGal and MTX were also able to significantly reduce the percentage of T-bet^+^/IFN-γ^+^ CD4^+^ T cells from RA after 18 h of incubation (Fig. 5C). We also tested IL-8 production by PBMC from RA patients and Control after GXMGal stimulation. Although IL-8 production was higher in RA than Control PBMC, the levels of this cytokine were not modulated neither by GXMGal nor MTX treatment at all times tested (Fig. 5D).

**Figure 6:**
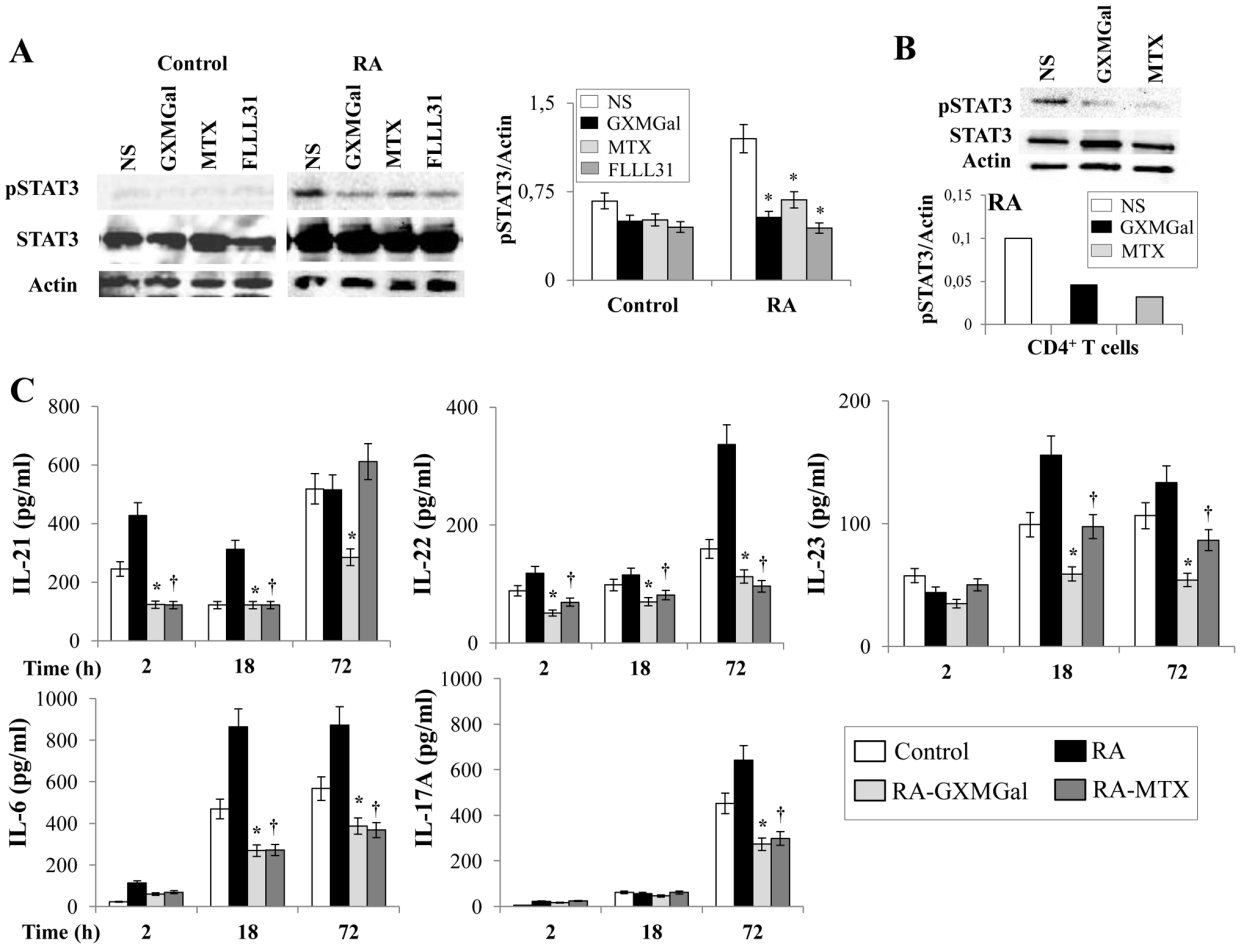
GXMGal effect on Th17 response. Activated PBMC (**A** and **C**) or purified CD4^+^ T cells (**B**) (both 5×10^6^/ml) from Control and RA were incubated for 2, 18 and 72 h in the presence or absence (NS) of GXMGal (10 µg/ml), MTX (10 ng/ml) or FLLL31 (5 µM). After 18 h of incubation, cell lysates were analyzed by western blotting. Membranes were incubated with Abs to pSTAT3 and STAT3. Actin was used as an internal loading control. Normalization was shown as mean ± SEM of five independent experiments (**A**) or as one representative experiment of three with similar results (**B**). *, *p*<0.05 (triplicate samples of 5 different Control and RA; RA treated *vs* untreated cells). Culture supernatants were collected after 2, 18 and 72 h to test IL-21, IL-22, IL-23, IL-6 and IL-17A levels by specific ELISA assays. *, *p*<0.05 (triplicate samples of 7 different Control and RA; RA GXMGal-treated *vs* untreated cells); ^†^, *p*<0.05 (triplicate samples of 7 different Control and RA; RA MTX-treated *vs* untreated cells) (**C**).

In addition to Th1 activation, Th17 cell activation plays a key role in the pathogenesis of arthritis [35]. To verify whether a down-regulation of Th17 occurs in our experimental condition after GXMGal treatment, we analyzed pSTAT3 activation, a master regulator of Th17 cells, by using PBMC from RA patients. FLLL31, a well-known inhibitor of STAT3 activation [36], was used as negative control. PBMC were stimulated with GXMGal and the pSTAT3 activator and Th17-related cytokines were tested. The results showed that pSTAT3 expression was down-regulated by GXMGal only in PBMC from RA patients, while no modulation was observed in PBMC from Control (Fig. 6A). Similar effects were observed after stimulation with FLLL31 or MTX (Fig. 6A). GXMGal-induced pSTAT3 inactivation at 18 h was also confirmed by using purified CD4^+^ T cells from RA (Fig. 6B). Subsequently, the production of cytokines involved in Th17 differentiation and activity were analyzed in PBMC from RA patients. Higher levels of IL-21 were produced by RA PBMC after 2 and 18 h with respect to Control; higher levels of IL-23 and IL-6 were produced by PBMC from RA patients after 18 and 72 h as compared to Control, while a significant increase of IL-22 and IL-17A was evidenced only after 72 h (Fig. 6C).

We observed an early inhibition (after 2 h) of IL-21 and IL-22 induced by GXMGal in PBMC from RA that persisted for 18 and 72 h. Moreover, the production of IL-6 and IL-23 was significantly inhibited after 18 and 72 h, while suppression of IL-17A was evidenced only after 72 h of GXMGal treatment (Fig. 6C). MTX showed the same kinetics of IL-22, IL-23, IL-6 and IL-17A inhibition than that observed for GXMGal, except for IL-21 after 72 h of incubation (Fig. 6C).

## Discussion

**Figure 7:**
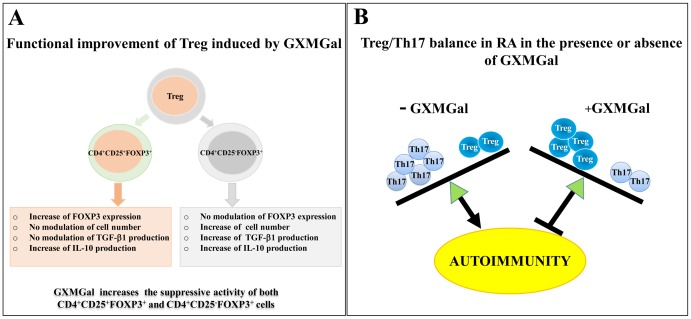
Schematic representation of GXMGal effects on Treg (A) and on Treg/Th17 balance (B).

FOXP3 is thought to be the main marker of Treg, since it plays a critical role in their development and maturation [37]. Compelling evidence show that FOXP3-deficient mice develop autoimmune disease [38], [39]. The role of Treg in RA has been partially elucidated and different results have been reported. In particular, a decreased number of Treg in the blood of RA patients has been observed [12]; however, other reports show high levels of circulating conventional CD4^+^CD25^+^FOXP3^+^ Treg cells in RA [13], [14], while additional studies reported an unaltered number [40]. Because of these controversial results about the number of circulating Treg cells, the interpretation of the data is problematic. Nevertheless, compelling evidence has emerged about the impaired function of these cells in RA patients [6]. Recent investigations revealed that a potentiation of Treg cells is beneficial in RA [40], [41]. In this study we report that the treatment with GXMGal induces strong and long-lasting increase of FOXP3 expression in RA Treg cells still evident 18 h after stimulation. This was accompanied by early and transient enhancement of TGF-β1 production and long-lasting production of IL-10. The major source of early production of TGF-β1 appeared to be the CD4^+^CD25^−^FOXP3^+^ T cell subset. On the contrary, the early production of IL-10 seems to be due to both CD4^+^CD25^+^FOXP3^+^ and CD4^+^CD25^−^FOXP3^+^ cells, although only CD4^+^CD25^+^FOXP3^+^ cells seemed to be responsible of its long-lasting production. We previously demonstrated that GXMGal inhibits pro-inflammatory cytokine secretion. Since the suppressive activity of Treg cells could be counteracted by inflammatory mediators [42], [43], particularly by TNF-α [44], it is possible that the increased activity of Treg cells induced by GXMGal could also include the previously described inhibition of TNF-α [1]. Accordingly, the treatment of RA subjects with an anti-TNF-α specific antibody was shown to restore Treg cell function via increased expression of FOXP3 phosphorylation [45]. Similarly, GXMGal could retain beneficial effects similar to those of anti-TNF-α treatment by influencing Treg cell activity. GXMGal did not modulate the percentage of CD4^+^CD25^+^FOXP3^+^ cells, while atypical CD4^+^CD25^−^FOXP3^+^ cells appeared to be numerically increased after GXMGal addition. The percentage of both cells was calculated on CD4^+^ T cells gated on PBMC, suggesting that the small and not statistically significant decline of CD4^+^CD25^+^FOXP3^+^, observed after GXMGal treatment, could at least in part account for the increased number of CD4^+^CD25^−^FOXP3^+^; however it is likely that besides the shift of CD4^+^CD25^+^FOXP3^+^ into CD4^+^CD25^−^FOXP3^+^, there is also an increase of the CD4^+^CD25^−^FOXP3^+^ cell number *per se*. Moreover, the significant increased expression of FOXP3 observed in conventional RA Treg cells after 18 h of GXMGal treatment clearly demonstrated that the associated increase of suppressive activity of all Treg is ascribed to CD4^+^CD25^+^FOXP3^+^ cells. GXMGal effects on different Treg subsets have been summarized in Figure 7.

MTX, widely accepted as the “gold standard” treatment in RA, can affect Treg cells [46] by promoting immunotolerance, despite the mechanisms are not yet fully elucidated. To our knowledge this is the first demonstration that MTX treatment increases the CD4^+^CD25^−^FOXP3^+^ fraction of the Treg cell population. Indeed, we found that the anti-inflammatory effects of GXMGal on T cells from RA were quite similar to that observed for MTX. GXMGal differed from MTX just in a few effects, such as increased production of IL-10 in atypical Treg. The fact that GXMGal retains multiple regulatory effects similar to that of MTX and anti-TNF-α agents suggests that GXMGal treatment may provide the combined beneficial effects of MTX and anti-TNF-α. Recently, we demonstrated that GXMGal inhibits Th17 activation [20] from RA patients and in the present study we confirm and extend our previously published data by demonstrating that the inhibition of Th17 was related to the inhibition of cytokines such as IL-21, IL-22 and IL-23. Moreover, we also provide evidence that in peripheral T cells from RA, GXMGal inhibits Th1 differentiation in terms of T-bet expression and IL-12p70 and IFN-γ production. Therefore, the demonstration of a Th1-cell suppression combined with a Th17-cell down-regulation reinforces the idea of a possible therapeutic use of GXMGal in RA and other inflammatory chronic disorders. GXMGal-treated Treg cells showed increased suppressive activity manifested by marked inhibition of effector T-cell proliferation. It is known that the total Treg cell pool contains a population of CD45RA^+^ and CD45RO^+^ Treg cells. CD45RA^+^ Treg cells were found to be less proliferative than their CD45RO^+^ counterparts [47]. In multiple sclerosis an impairment of suppressive activity of naïve CD45RA^+^ Treg cells was reported, thereby suggesting that this population may be involved in the pathogenesis of autoimmune disorders [48], [49]. GXMGal is able to bind to CD45RA and CD45RO isoforms and we recently suggested that activated T cells expressing the CD45RO molecule could be the main target of GXMGal-induced apoptosis [20]. Since the Treg population circulating in RA belongs to (naïve) resting CD45RA phenotype [50], it is conceivable that GXMGal binds to these cells and increases their function without inducing apoptosis. Therefore these results suggest that in RA patients GXMGal affects Treg function in a two ways: indirectly by inhibition of inflammatory T cells differentiation such as Th1 and Th17 and directly by binding to CD45RA improving activity of Treg. In this study the effect of GXMGal was specifically observed on Treg population as demonstrated by: i) enhanced production of TGF-β1, ii) increased expression of FOXP3 and iii) improved suppression activity on effector T cells. Future studies will be devoted to evaluate the effect of GXMGal in an *in vivo* experimental model of rheumatoid arthritis. Collectively these data highlight the potential for using GXMGal to enhance Treg activity, inactivate pathogenic T cells and halt the disease process in autoimmunity.
