# Expression of Concern: Functional Improvement of Regulatory T Cells from Rheumatoid Arthritis Subjects Induced by Capsular Polysaccharide Glucuronoxylomannogalactan

**DOI:** 10.1371/journal.pone.0247971

**Published:** 2021-02-25

**Authors:** The PLOS ONE Editors

Following publication of this article [1], concerns were raised about duplication of western blot images in Figs 1 and 6. Specifically,

- In Fig 1A the 2 h Actin panel for Control cells is a duplicate of the first three lanes of the Actin panel for Control cells in Fig 6A.

- In Fig 6B the Actin panel is incorrect and is a duplicate of the 18 h Actin panel for RA cells in Fig 6A of another *PLOS ONE* article [2] reporting a different experiment.

The corresponding author has stated that there were errors in selection of western blot images for Figs 1 and 6 as follows:

- In the originally published Fig 1A, the panels for Control cells are not taken from the same replicate experiment as the panels for the RA cells. A revised Fig 1 is provided in which all panels showing results for Control cells in 1A are replaced such that the revised figure shows the results for Control and RA cells from the same experiment.

**Fig 1:**
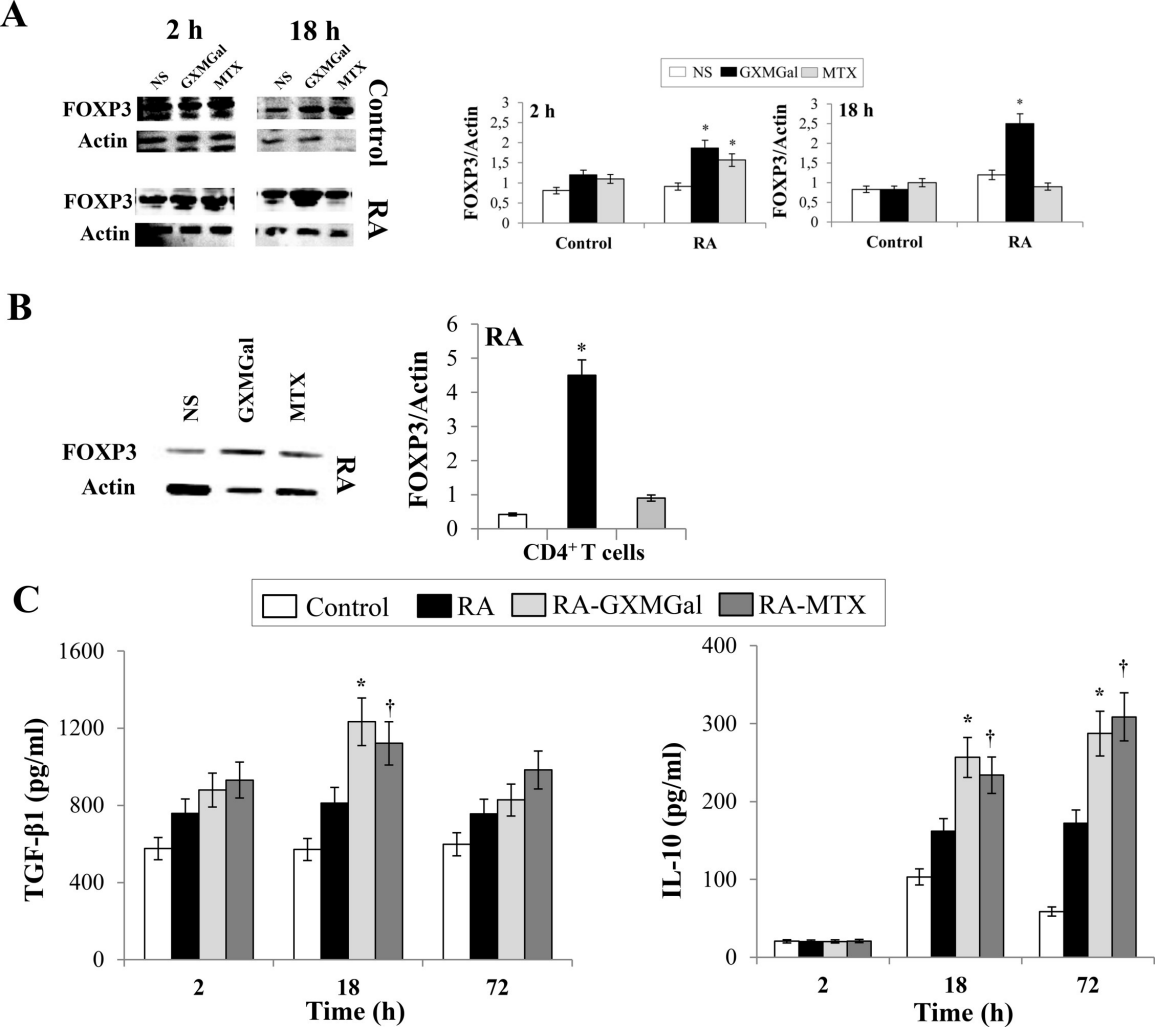
GXMGal effect on Treg cell response. Activated PBMC (**A** and **C**) or purified CD4^+^ T cells (**B**) (both 5×10^6^/ml) from Control and RA were incubated for 2, 18 and 72 h in the presence or absence (NS) of GXMGal (10 μg/ml) or MTX (10 ng/ml). After 2 and 18 h (**A**) or 18 h (**B**) of incubation, cell lysates were analyzed by western blotting. Membranes were incubated with Ab to FOXP3. Actin was used as an internal loading control. Normalization was shown as mean ± SEM of five independent experiments (**A** and **B**). *, *p*<0.05 (triplicate samples of 5 different Control and RA; RA treated *vs* untreated cells). Note that for both Control and RA cells the immunoblots at 18 h (**A**) share Actin loading control panels with the experiment shown in Fig 6A, as the same immunoblots were stripped and re-probed using different antibodies. Culture supernatants were collected after 2, 18 and 72 h to test TGF-β1 and IL-10 levels by specific ELISA assays. *, *p*<0.05 (triplicate samples of 7 different Control and RA; RA GXMGal-treated *vs* untreated cells); †, *p*<0.05 (triplicate samples of 7 different Control and RA; RA MTX-treated *vs* untreated cells) (**C**).

- In the originally published Fig 6A, the panels for Control cells are not taken from the same replicate experiment as the panels for the RA cells. Additionally, the pSTAT3 and Actin panels for Control cells are incorrect and were taken from blots carried out at an earlier time point than stated (2 h as opposed to 18 h). In the originally published Fig 6B, the Actin panel is incorrect and was taken from a different experiment. A revised Fig 6 is provided in which all panels showing results for Control cells in 6A are replaced such that the revised figure shows the results for Control and RA cells from the same experiment, all carried out at the 18 h timepoint. The incorrect Actin panel in 6B is also replaced with the correct corresponding Actin loading control for this experiment. The revised Fig 6 also addresses an error in the y-axis of normalized graphs in parts 6A and 6B, which are corrected to show that pSTAT3 was normalized against STAT3.

**Fig 6:**
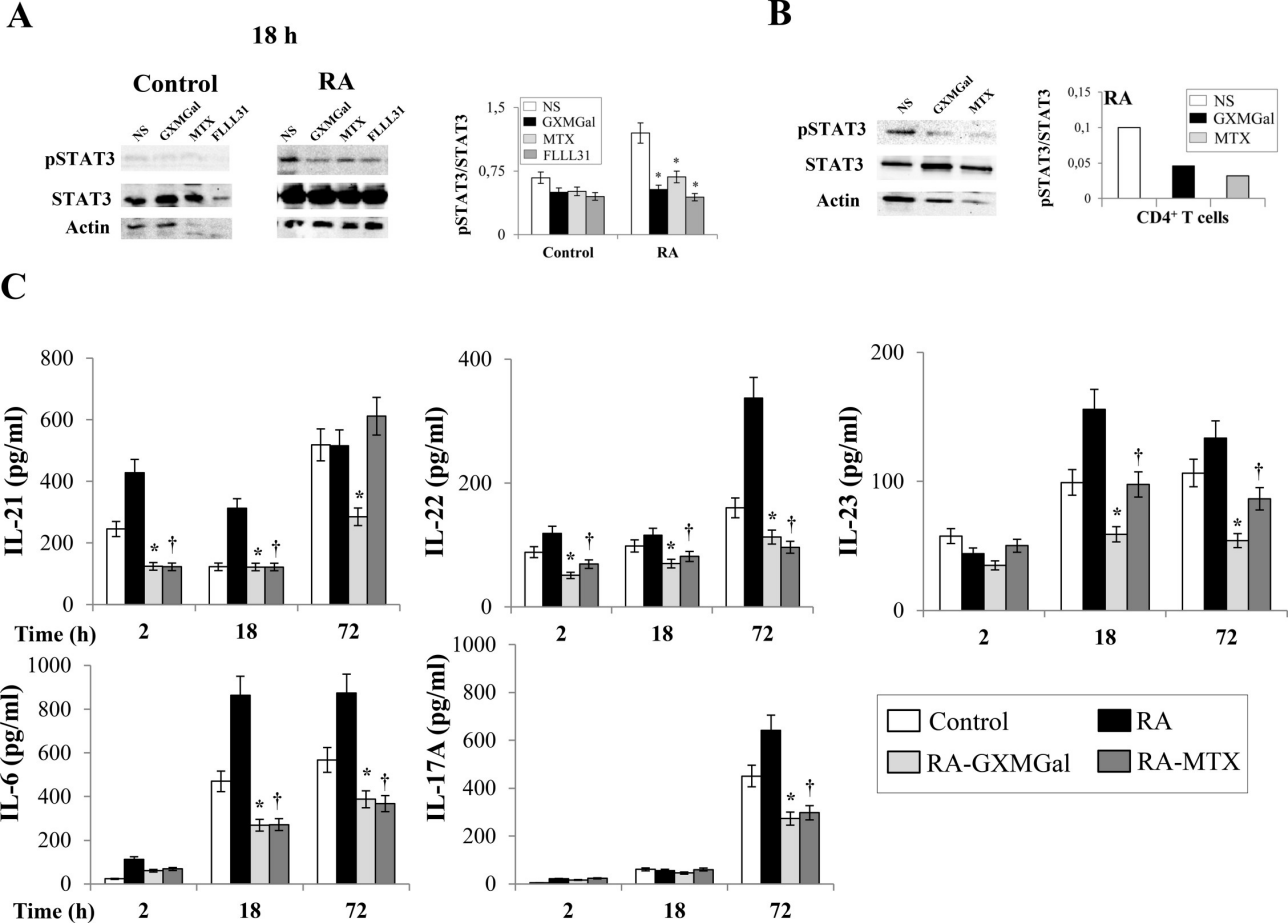
GXMGal effect on Th17 response. Activated PBMC (**A** and **C**) or purified CD4^+^ T cells (**B**) (both 5×10^6^/ml) from Control and RA were incubated for 2, 18 and 72 h in the presence or absence (NS) of GXMGal (10 μg/ml), MTX (10 ng/ml) or FLLL31 (5 μM). After 18 h of incubation, cell lysates were analyzed by western blotting. Membranes were incubated with Abs to pSTAT3 and STAT3. Actin was used as an internal loading control. Normalization was shown as mean ± SEM of five independent experiments (**A**) or as one representative experiment of three with similar results (**B**). *, *p*<0.05 (triplicate samples of 5 different Control and RA; RA treated *vs* untreated cells). Note that for both Control and RA cells in (**A**), the immunoblots share Actin loading control panels with the experiments shown in Fig 1A at 18 h, as the same immunoblots were stripped and re-probed using different antibodies. Culture supernatants were collected after 2, 18 and 72 h to test IL-21, IL-22, IL-23, IL-6 and IL-17A levels by specific ELISA assays. *, *p*<0.05 (triplicate samples of 7 different Control and RA; RA GXMGal-treated *vs* untreated cells); †, *p*<0.05 (triplicate samples of 7 different Control and RA; RA MTX-treated *vs* untreated cells) (**C**).

The corresponding author has clarified that in revised Figs 1A and 6A for both Control and RA cells, the 18 h immunoblot is the same blot stripped and re-probed with different antibodies, thus the Actin loading control is expected to be the same. The revised figure legends include a statement to clarify this.

The available underlying western blot image files for this study are provided as Supporting Information files. They can be viewed below. The original uncropped image files for western blots underlying Figs 1B Actin panel, 6A RA Actin panel, 6A Control STAT3 and pSTAT3 panels, and all panels of 6B are unavailable. The original flow cytometry data files underlying Fig 2A, 3A, 4A are unavailable.

The corresponding author states that the errors occurred during figure assembly and did not affect any of the associated quantitative data or reported conclusions.

Editorial assessment in consultation with a member of the Editorial Board identified a concern that the representative blots for FOXP3 expression in Control cells at 18h in the revised Fig 1A appear to show higher levels of FOXP3 after MTX treatment compared with NS, in contrast with the quantitative data shown in the accompanying chart which indicates similar expression levels for all treatments for control cells at 18h. The corresponding author has stated that the difference is accounted for because the densitometry of 18h control in Fig 1A (right panel) is the mean of 5 independent western blot experiments. Additionally, it was noted that in the replacement blots for Figs 1A, 6A and 6B, the control cells show a lower signal for actin under MTX treatment for 18 h, and a similarly lower actin signal under FLLL31 treatment for 18 h in Fig 6A, raising a question over the function of actin as a loading control for this experiment and whether STAT3 levels, against which pSTAT3 levels are normalised in these figures, are consistent across treatments. The underlying blots for the other western blot replicates and the individual-level densitometry data underlying the charts in Figs 1 and 6 are no longer available to inform the editorial assessment of these issues.

The *PLOS ONE* Data Availability Policy requires that, with few exceptions, all data underlying the findings described in an article are fully available without restriction. While looking into the image issues raised, it came to the attention of the *PLOS ONE* Editors that the individual-level data underlying the charts in all figures cannot be made available in accordance with the above policy.

The *PLOS ONE* Editors issue this Expression of Concern to alert readers of these concerns about the unavailability of a number of underlying data files. It is additionally noted that in the absence of several original blot images and the individual-level densitometry data, the *PLOS ONE* Editors are unable to fully resolve queries regarding interpretation of the western blot data.

There is an update to the corresponding author’s contact information for this article. The corresponding author’s current email address is vecchiar10@gmail.com.

## Supporting information

S1 File Underlying blots for the replacement panels in Fig 1A (all Control panels) and in Fig 6A (Actin Control panel). (PDF)

S2 File Underlying blots for Fig 1A RA FOX-P3, 1B FOX-P3, Fig 5A (all panels), Fig 6A RA pSTAT3 and STAT3. (PDF)
