# Correction to Expression of Concern on: Functional Improvement of Regulatory T Cells from Rheumatoid Arthritis Subjects Induced by Capsular Polysaccharide Glucuronoxylomannogalactan

**DOI:** 10.1371/journal.pone.0249512

**Published:** 2021-03-29

**Authors:** PLOS ONE Staff

## Notice of republication

This Expression of Concern [1] was republished on March 5, 2021, to correct an error in the figure mentions. Specific figure mentions were erroneously hyperlinked during the typesetting process. Additionally, an incorrect version of the corresponding author’s email was published in error. The publisher apologizes for the error. Please download this Expression of Concern again to view the correct version. The originally published, uncorrected notice and the republished, corrected notice are provided here for reference.
