# Supplementary material for: Expression of Concern: Functional Improvement of Regulatory T Cells from Rheumatoid Arthritis Subjects Induced by Capsular Polysaccharide Glucuronoxylomannogalactan
Source: PLoS One. 2021 Feb 25;16(2):e0247971. doi: 10.1371/journal.pone.0247971 (PMC7906360; doi:10.1371/journal.pone.0247971)

Fig 1A FOXP3 Western blot.jpeg (Control 2 h and 18 h)

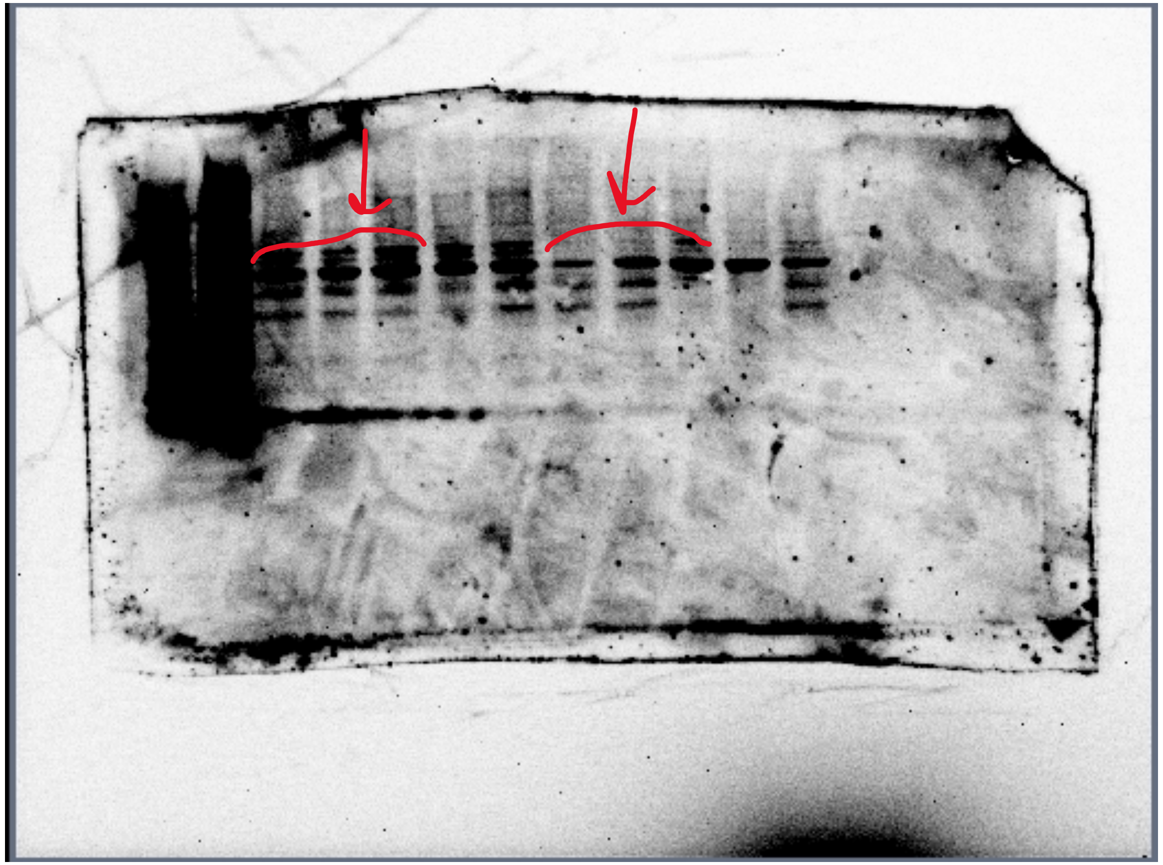

Fig 1A Actin Western blot.jpeg (Control 2 h and 18 h)

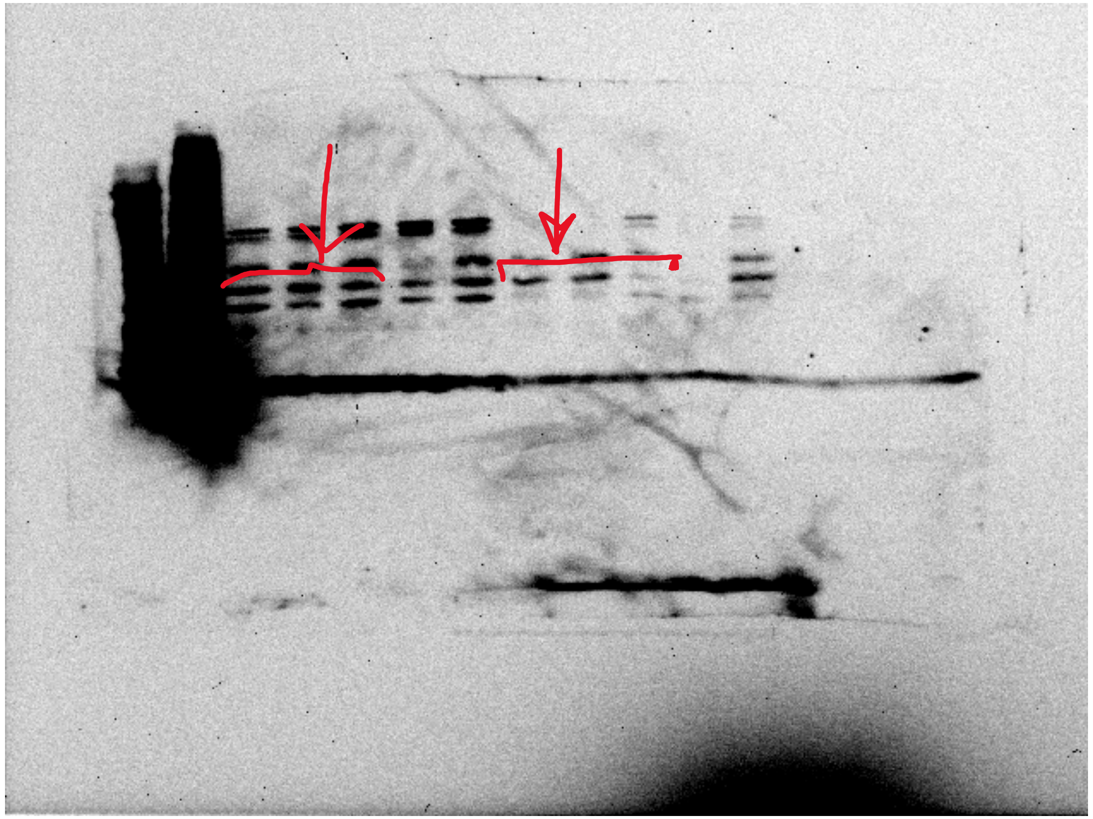

Fig 6A Actin Western blot.jpeg (Control 18h)

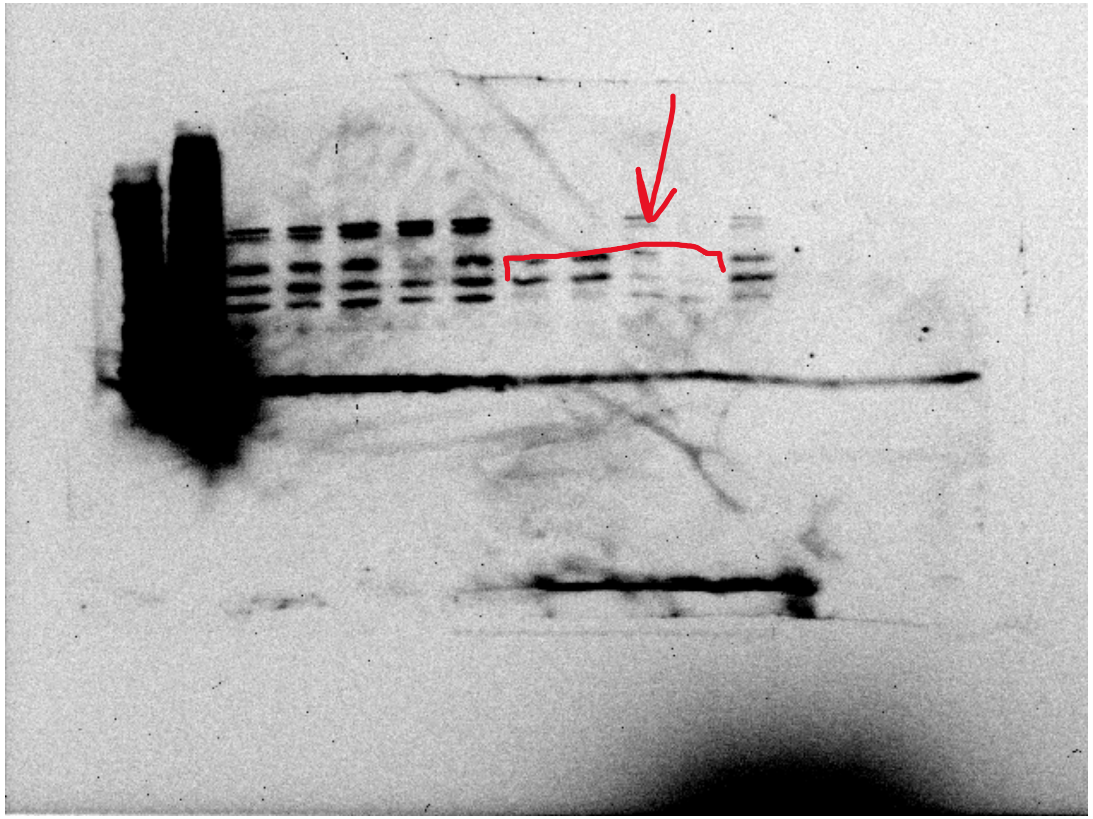

Supplement: S1 File — (PDF) [file pone.0247971.s001.pdf]
